# Berry anthocyanidins inhibit intestinal polyps and colon tumors by modulation of Src, EGFR and the colon inflammatory environment

**DOI:** 10.18632/oncoscience.548

**Published:** 2021-12-10

**Authors:** Ashley M. Mudd, Tao Gu, Radha Munagala, Jeyaprakash Jeyabalan, Mostafa Fraig, Nejat K. Egilmez, Ramesh C. Gupta

**Affiliations:** ^1^Department of Pharmacology and Toxicology, University of Louisville, Louisville, KY 40202, USA; ^2^Department of Microbiology and Immunology, University of Louisville, Louisville, KY 40202, USA; ^3^James Graham Brown Cancer Center, University of Louisville, Louisville, KY 40202, USA; ^4^Department of Medicine, University of Louisville, Louisville, KY 40202, USA; ^5^Department of Pathology and Laboratory Medicine, University of Louisville, Louisville, KY 40202, USA; ^*^These authors contributed equally to this work

**Keywords:** anthocyanidins, bilberry, colorectal cancer, familial adenomatous polyposis, PD-L1

## Abstract

Colorectal cancer is the third most common form of cancer diagnosed and the third leading class for cancer-related deaths. Given the prevalence of colon cancer worldwide, further insight into developing novel and effective prevention and treatment strategies are warranted. The family of plant pigments known as the anthocyanins has been identified with a variety of health benefits including chemopreventive and therapeutic effects. A limitation to current clinical applications of anthocyanins is the high doses that are required. In order to overcome this limitation, we tested the active moiety, anthocyanidins for chemopreventive and therapeutic effects against colorectal cancer *in vivo* and *in vitro*. Treatment with native anthocyanidin mixture (Anthos) from bilberry yielded significant antiproliferative activity against colon cancer cells. Anthos treatment led to significant reductions in polyp and tumor counts *in vivo*. Reduced Src and EGFR phosphorylation was observed with Anthos treatment, which correlated with downstream targets such as PD-L1 and modulation of the colon inflammatory environment. These results provide a promising outlook on the impact of berry Anthos for the treatment and prevention of familial adenomatous polyposis and colorectal cancer. Results from this study also provide novel mechanistic insight into the chemopreventive and therapeutic activities of Anthos.

## INTRODUCTION

Colorectal cancer (CRC) is the third leading type of cancer diagnosed and third leading cause of cancer-related deaths within the United States each year. According to the American Cancer Society, an estimated 104,270 individuals will be diagnosed and 45,230 will die from CRC in the U.S. alone in 2021. Although the overall incidence of developing CRC has been decreasing since the mid to late 1980s for individuals over 55-years old, a recent study has uncovered a disquieting increase in the CRC incidence for individuals below 40 years [1]. Although much progress has been made in combating the disease due to advancements made in early detection of CRC, clinically-effective chemopreventive measures are warranted.

The majority of CRC cases originate from previously benign adenomatous polyps [2]. This process of transformation from benign polyp to malignancy typically takes decades to occur, with approximately 85% of CRC cases occurring after the age of 55 years, according to data acquired by the U.S. preventive services task force. A small subset of CRC cases stem from familial syndromes. One such precondition for colorectal cancer is familial adenomatous polyposis (FAP). FAP is an autosomal dominant pre-cancerous colorectal condition with an incidence at birth of around 1/8,300 [3] that is caused by mutation(s) in the adenomatous polyposis coli (APC) gene. In FAP patients, EGFR has been found to be upregulated in most adenomas and carcinomas, with associated upregulation of downstream members of the signaling pathway [4]. FAP occurs with equal frequency in both males and females [3]. The disease symptoms typically become evident as early as the teenage years. Approximately 7% of individuals with FAP will develop CRC by the time they reach 21 years and 95% of FAP sufferers will develop CRC by the time they reach the age of 50 [5]. The only currently approved standard of care for the treatment of FAP is surgical resection of the colon [6]. Even after removal of the colon, individuals with FAP are still at risk for developing adenomas at other sites [6]. Given the limited treatment options for FAP and need for clinically-effective chemopreventive measures for CRC, there is a need for additional research for more effective treatment and prevention methods.

A wide variety of compounds have been considered in both pre-clinical and clinical populations for the prevention of CRC and/or FAP. Clinical trials conducted with chemopreventive agents for CRC include non-steroidal anti-inflammatory drugs such as aspirin [7] and the cyclo-oxygenase-2 (COX-2) inhibitors, calcium [8], vitamin D [9], folic acid [10], and dietary phenolics such as resveratrol [11], curcumin [12], green tea extract [13], soy isoflavones [14], black raspberry powder [15], pomegranate extract [16] and bilberry extract [17]. However, the application of these interventions either were deemed ineffective, required large dosages or presented undesired side effects [18, 19]. Therefore, identification of plant bioactives present in the human environment for improved clinical translation is of utmost importance.

One particular class of compounds, the anthocyanins, have recently been of great interest in the scientific and medical community due to their reported therapeutic activities including anti-inflammatory [20], chemopreventive and chemotherapeutic [21, 22], obesity control, cardiovascular disease prevention, and alleviation of the symptoms of diabetes [23]. Anthocyanins are ubiquitous plant pigments that provide the characteristic red, purple and blue hues in vegetables (eggplant, purple cabbage), fruits (berries), grains (black rice, purple corn) and flowers (hibiscus). In fact, over 600 anthocyanins have been identified at the current time [24]. Anthocyanins feature an aryl-substituted chroman ring system. Differences between each specific anthocyanin is dependent upon the specific functional group(s) at the 3 and 5 prime locations and the composition of the pendant sugars. Furthermore, the active moiety of anthocyanins, anthocyanidins are devoid of the sugar residues but feature the same key central ring structure and various permutations of hydroxyl, hydrogen and methoxy functional groups.

Studies from our laboratory as well as others have shown therapeutic activity of anthocyanins against a variety of cancers including but not limited to breast, lung, esophageal, skin and CRC [22, 25]. One such study by Cooke et al. [26] showed that ingestion of cyanidin-3-glucoside or a bilberry-derived enriched anthocyanin mixture showed a modest reduction in adenoma in CRC in a dose-dependent fashion. This, however, required a rather large dose (450 mg/kg/day). When the native bilberry extract (Mirtoselect) was extrapolated to the amounts of fresh berries that would need to be consumed, it was concluded that an individual would need to consume ~740 g of fresh bilberry a day. Given the large nature of the dose used in the study, which is further complicated by the presence of high sugar content in berries, it is clear that one would need to lower the dose by identifying the active principles for clinical translatability. Previous work from our group has shown that anthocyanidins yield greater potency *in vitro* and *in vivo* against lung cancer [27, 28]. The goal of the series of experiments contained within this manuscript was to elucidate whether Anthos would be an efficacious treatment in preventing and treating FAP and CRC in a clinically-relevant bacterially-induced murine model and in identifying potential mechanisms of action pertaining to the role of Anthos in modulation of Src and EGFR pathways and modulation of the inflammatory environment.

## RESULTS

### Anti-proliferative effects of Anthos on colon cancer cells but not normal colon cells

**Figure 1:**
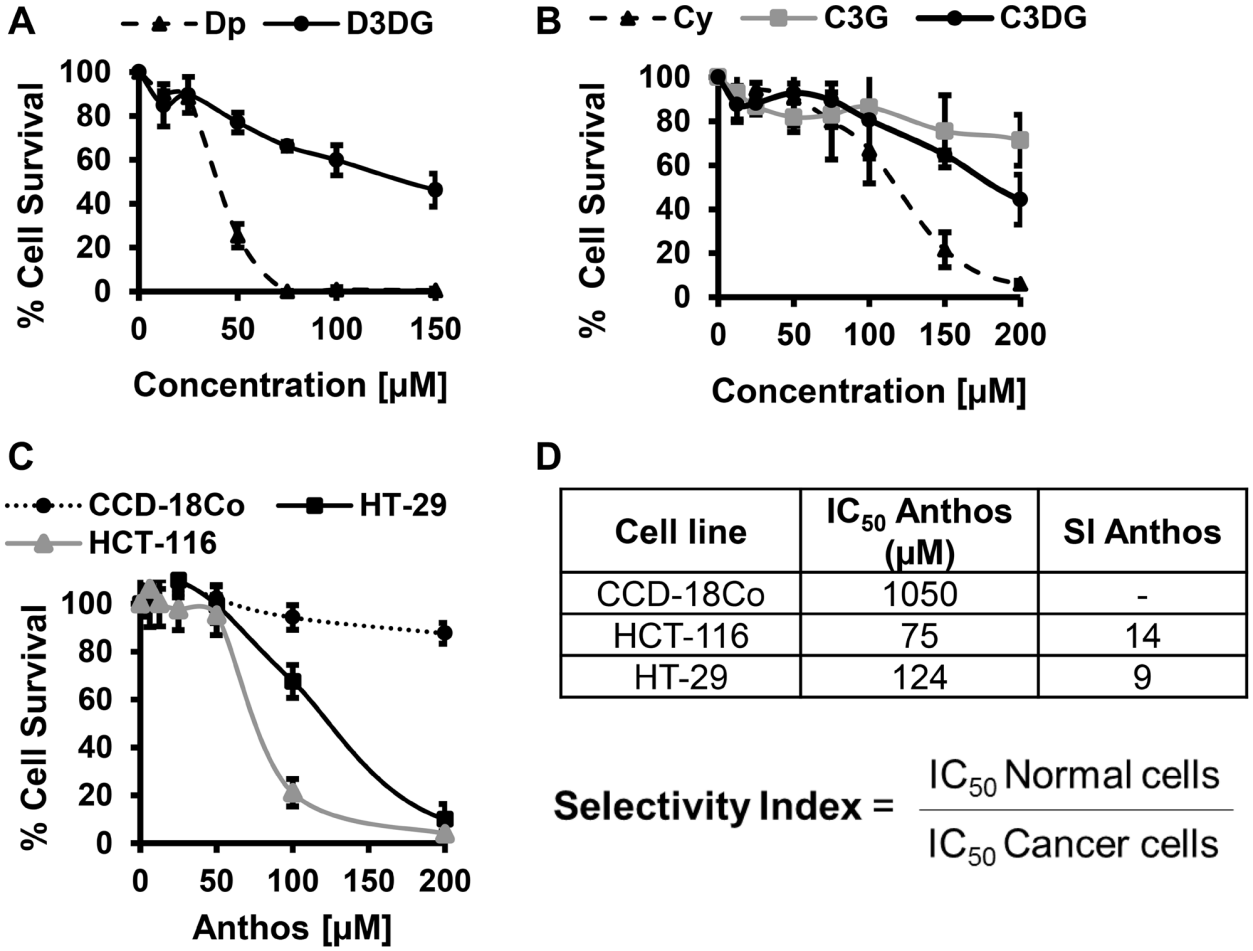
Antiproliferative activity of select anthocyanidins and their anthocyanin counterparts against colon cancer cell line HCT-116 and the antiproliferative activity of bilberry-derived anthocyanidin mixture (Anthos) against colon normal and cancer cells *in vitro*. Colon cancer HCT 116 cells were treated with various concentrations of the indicated anthocyanidins and anthocyanins for 72 h and the effect on cell growth inhibition was assessed using an MTT assay (**A** and **B**); data represent average ± SD (*n* = 3). Colon normal cells, CCD-18Co and colon cancer cells, HCT-116 and HT-29 were treated with various concentrations of Anthos for 72 h and the effect on cell growth inhibition was assessed using an MTT assay (**C**); data represent average ± SEM (*n* = 4). (**D**) Table with tabulated selectivity index values for Anthos in colon normal vs. cancer cells *in vitro*.

Previous work within our laboratory has shown that anthocyanidins are more potent than their respective anthocyanins [27]. However, prior to pursuing work with Anthos, we first compared antiproliferative activity of anthocyanidins and anthocyanins by using the individual anthocyanins (delphinidin 3,5-diglucoside, cyanidin 3,5-diglucoside and cyanidin 3-glucoside) with their respective anthocyanidins (delphinidin and cyanidin) in HCT-116 colon cancer cells. These particular anthocyanins were selected due to their presence in the native bilberry mixture. As shown in Figure 1A and 1B, cleavage of the sugar moiety from the anthocyanin parent compound led to reduced IC_50_ values in colon cancer cells. This is best demonstrated by the reduction in the IC_50_ for delphinidin which yielded >6-fold reduction when the sugar moiety was absent. A reduction in the IC_50_ was also observed when comparing cyanidin versus cyanidin 3-glucoside, and cyanidin 3,5-diglucoside, with 2- and nearly 5-fold reductions, respectively.

We then assessed the antiproliferative properties of Anthos isolated from bilberry against colon cancer cells (HCT-116 and HT-29) and normal colon cells (CCD-18Co) (Figure 1C and 1D). Tabulated selectivity index (SI) values (Figure 1D) of 14 and 9 clearly show that Anthos selectively target colon cancer cells over the normal cells. It should be noted that these values are well above the recommended minimal SI value of 3 that is commonly used to determine whether a anti-cancer drug selectively targets cancer cells over normal cells [29].

### Impact of Anthos on polyp development in *Apc^Min^*^/+^ mouse FAP model

**Figure 2:**
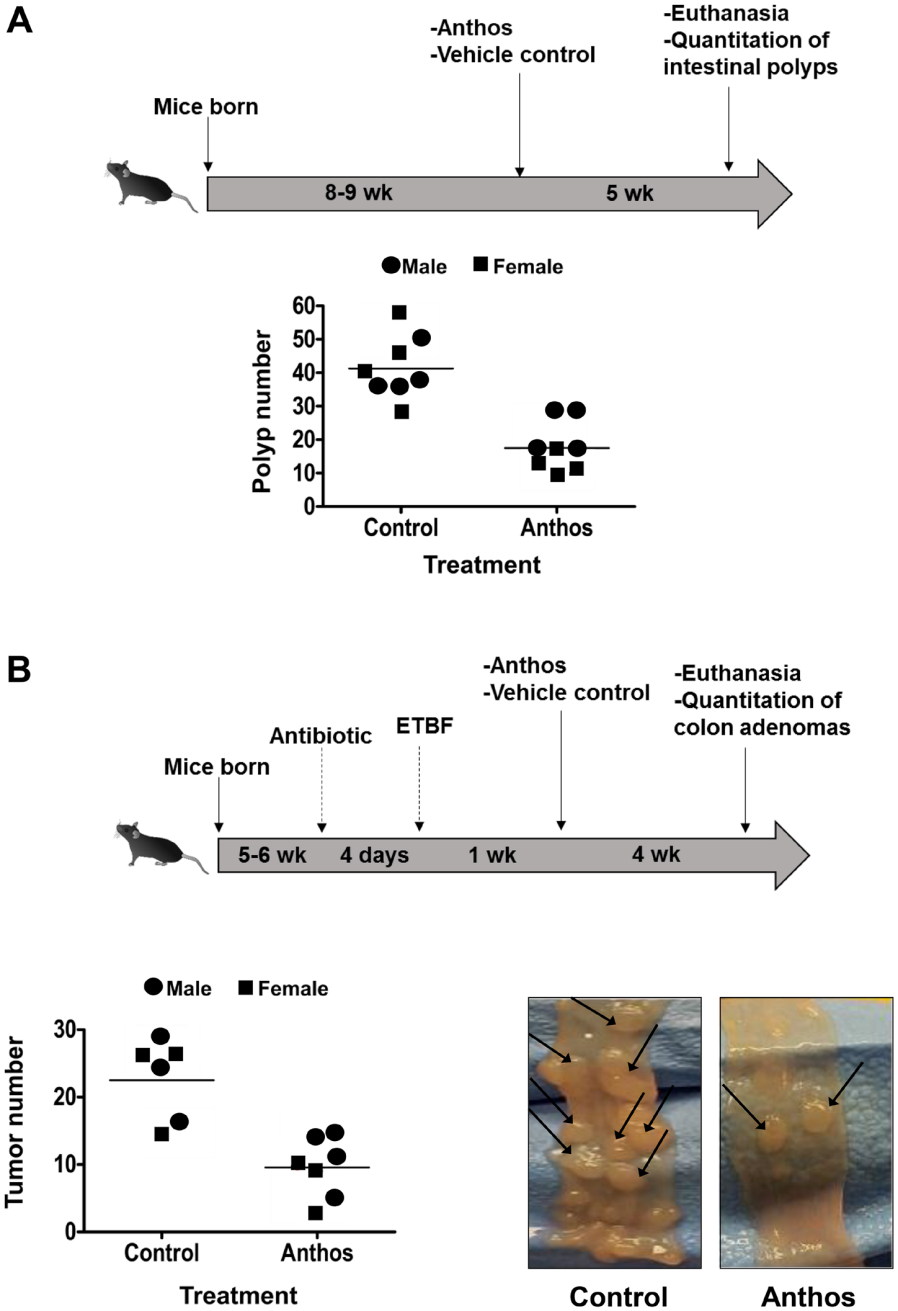
Anti-polyp and anti-tumor activities of Anthos against intestinal polyps and colon tumors. (**A**) *Apc^Min^*^/+^ mice were treated via oral gavage with bilberry-derived anthocyanidin mixture (Anthos) three times a week (40 mg/kg bw) or vehicle control. Data represent the distribution of animal polyp counts by gender, with the average noted. Male mice, Anthos versus control *P* = 0.02 and female mice, Anthos versus control *P* = 0.004. (**B**) *Apc^Min^*^/+^ mice inoculated with ETBF were treated via oral gavage with Anthos three times per week (40 mg/kg bw), five times per week (20 mg/kg bw), or vehicle control. Data represent the distribution of animal tumor counts by gender, with the average noted. Male and female mice, Anthos versus control *P* < 0.001. Representative images of colons taken from control versus Anthos-treated animals, with arrows highlighting tumors.

A wide variety of plant-derived compounds, including but not limited to curcumin, epigallocatechin gallate and anthocyanins have been shown to have chemopreventive properties [19]. However, large doses have been a hallmark prerequisite for their efficacy *in vivo* [19]. Therefore, in order to test whether Anthos are both efficacious and translatable for the required dose to elicit such properties, we investigated the effect of the berry Anthos on intestinal polyp number utilizing *Apc^Min^*^/+^ mice. Both male and female mice (*n* = 4 per group) were treated with 40 mg/kg Anthos three times per week (≈17 mg/kg/day) for four weeks by oral gavage. Results from the study (Figure 2A) showed a 2-fold reduction in intestinal polyps with the Anthos treatment compared to vehicle control. The results in both male and female mice treated with the Anthos compared to the vehicle control were statistically significant (*P* = 0.02 and *P* = 0.004, respectively). Interestingly, when the data was stratified by sex, it was noted that the effect of Anthos treatments was somewhat enhanced in female mice which showed 3.1 fold reductions in polyp numbers (*P* = 0.004) as compared to the 1.8 fold reduction (*P* = 0.02) found within males.

### Impact of Anthos on colon tumor development in enterotoxigenic *B. fragilis* (ETBF) *Apc^Min^*^/+^ mice CRC model

**Figure 3:**
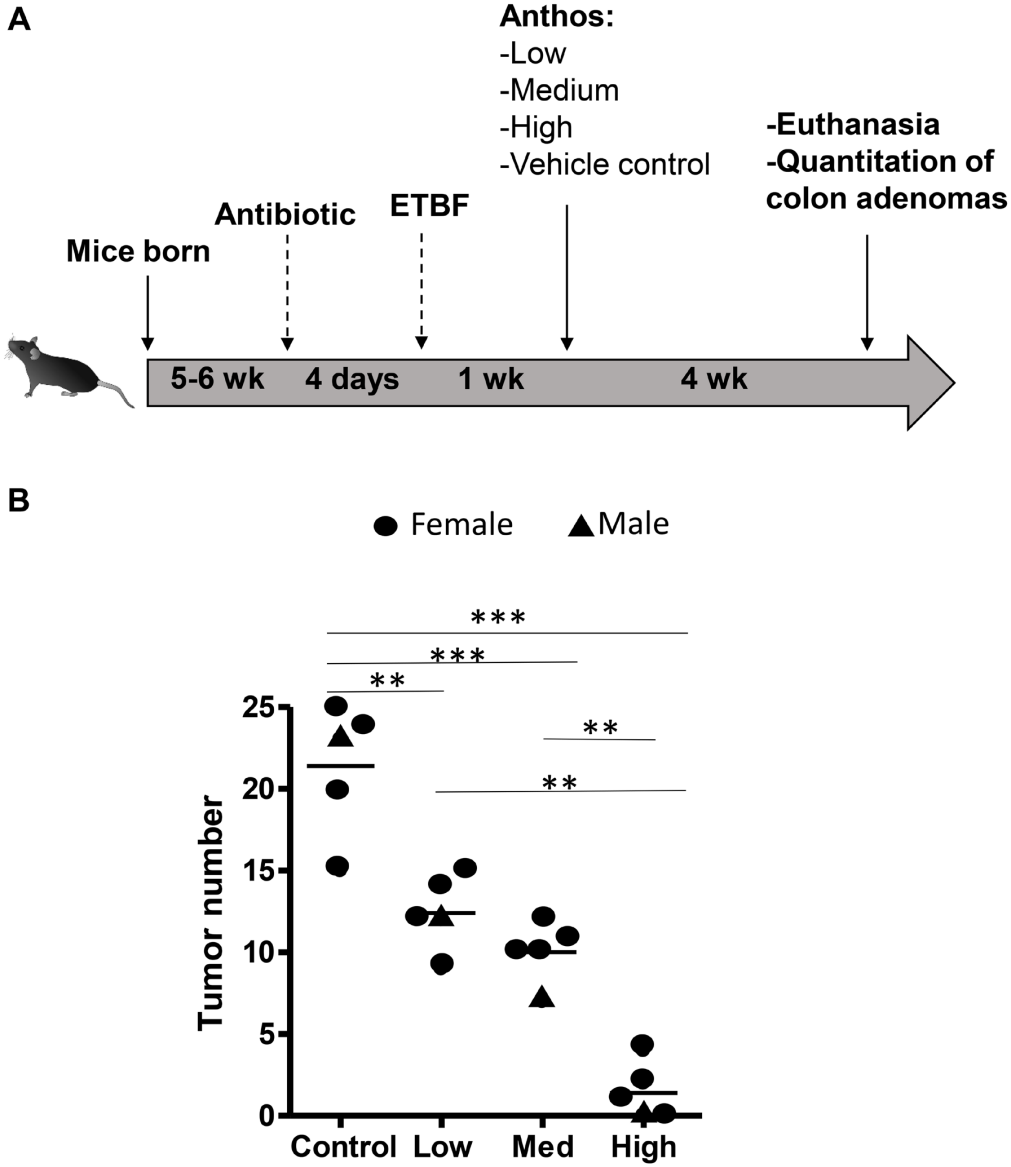
*In vivo* Anthos dose escalation study. *Apc^Min^*^/+^ mice inoculated with ETBF were treated via oral gavage for 4 weeks with low (≈8.6 mg/kg/day), medium (≈17.1 mg/kg/day), high (≈34.3 mg/kg/day) dosages or vehicle control. (**A**) Study overview. (**B**) tumor numbers. Black triangles represent males and black circles represent females. ^***^signifies *P* < 0.001, ^**^signifies *P* < 0.01, and ^*^signifies *P* < 0.05.

Previous work within our group has shown that Anthos decrease tumor burden in lung and ovarian cancer models [28, 30]. However, no work has been reported to assess the impact of bilberry-derived Anthos against either FAP, colorectal cancer or bacterially-induced cancers. Therefore, we investigated whether Anthos treatment would impact colon tumor development in a bacterially-driven *Apc^Min^*^/+^ colorectal cancer mouse model. In these studies, mice which were treated with Anthos at either 20 mg/kg Anthos 5 times per week (equivalent to daily dose of 14 mg/kg) or 40 mg/kg 3 times per week (equivalent to daily dose of 17 mg/kg) for 4 weeks by oral gavage showed an average reduction of 2.6 fold in the number of colorectal tumors in Anthos-treated mice compared with control (Figure 2B). This reduction was statistically significant (*P* < 0.001). In the subsequent dose-escalation study, three doses were tested equivalent to- low (20 mg/kg, three doses a week, ≈8.6 mg/kg/day), medium (40 mg/kg, three doses a week, ≈17.1 mg/kg/day) and high (80 mg/kg, three doses a week, ≈34.3 mg/kg/day). Controls included a vehicle control and an *Apc^Min^*^/+^ mice without ETBF bacteria control. Results from this study (Figure 3) showed a clear dose-dependent decrease in colon tumor counts in Anthos-treated groups. Significant differences in tumor counts were noted between control and low dose animals (*P* = 0.003), control and medium dose animals (*P* = 0.0004), control and high dose animals (*P* < 0.0001). Additionally, significant inter-treatment group differences were noted for medium and high doses (*P* = 0.0001) and low and high doses (*P* < 0.0001). The difference between low and medium treatment groups was border-line significant (*P* = 0.054). Number of animals in this study was too small to assess gender-related differences.

### Influence of Anthos on tumor grade and immune response

**Figure 4:**
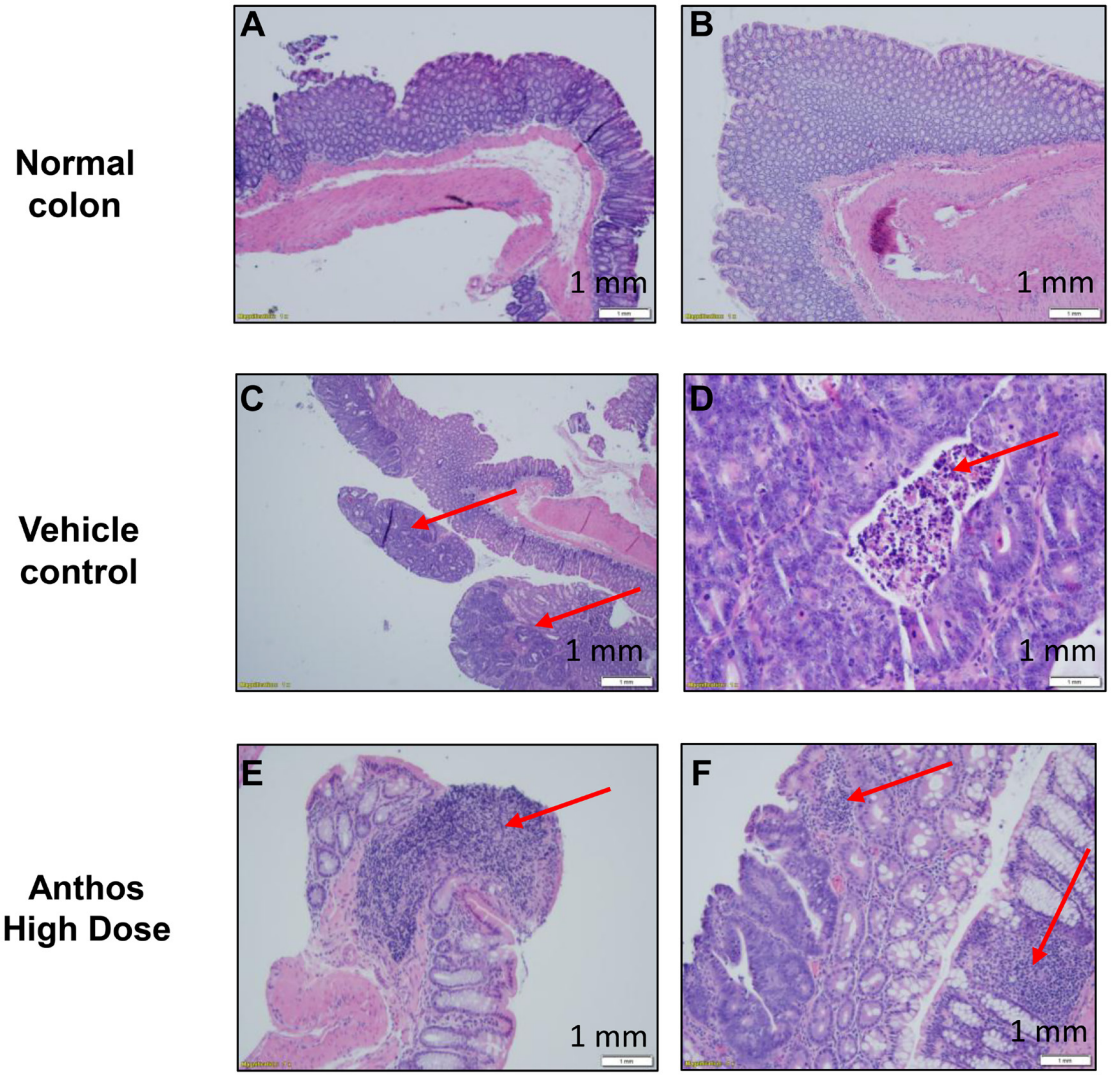
Anthos-treated *Apc^Min/+^* mice inoculated with ETBF featured fewer, more well differentiated tumors, with immune response. Representative images of histological analysis of colon adjacent normal and tumor samples taken from normal colon (**A**, **B**), vehicle control (**C**, **D**) and Anthos-treated (**E**, **F**). Paraffin sections of colons were stained with H&E. Arrows in C and D highlight representative tumors (C) and necrotic patches (D) observed in vehicle control mice. Arrows in E and F represent lymphoid aggregates adjacent to normal (E) and tumor and normal colon tissue (F). No lymphoid aggregates were observed in vehicle control mice. Scale bars = 1 mm for all figure panels.

Tumor and adjacent normal tissue samples from the Anthos dose-escalation study underwent histopathological analysis (Figure 4A–4F). Results from this analysis showed that Anthos-treated animals tended to have more well-differentiated, smaller tumors than the vehicle treatment which tended to have larger more moderately-differentiated tumors (Figure 4C and 4D). Additionally, Anthos-treated animals were found to have lymphoid aggregates in the adjacent normal and abnormal tissue (tumor + normal) (Figure 4E and 4F). No lymphoid aggregates were found in the control animals.

### Insights into Anthos mechanism of action

**Figure 5:**
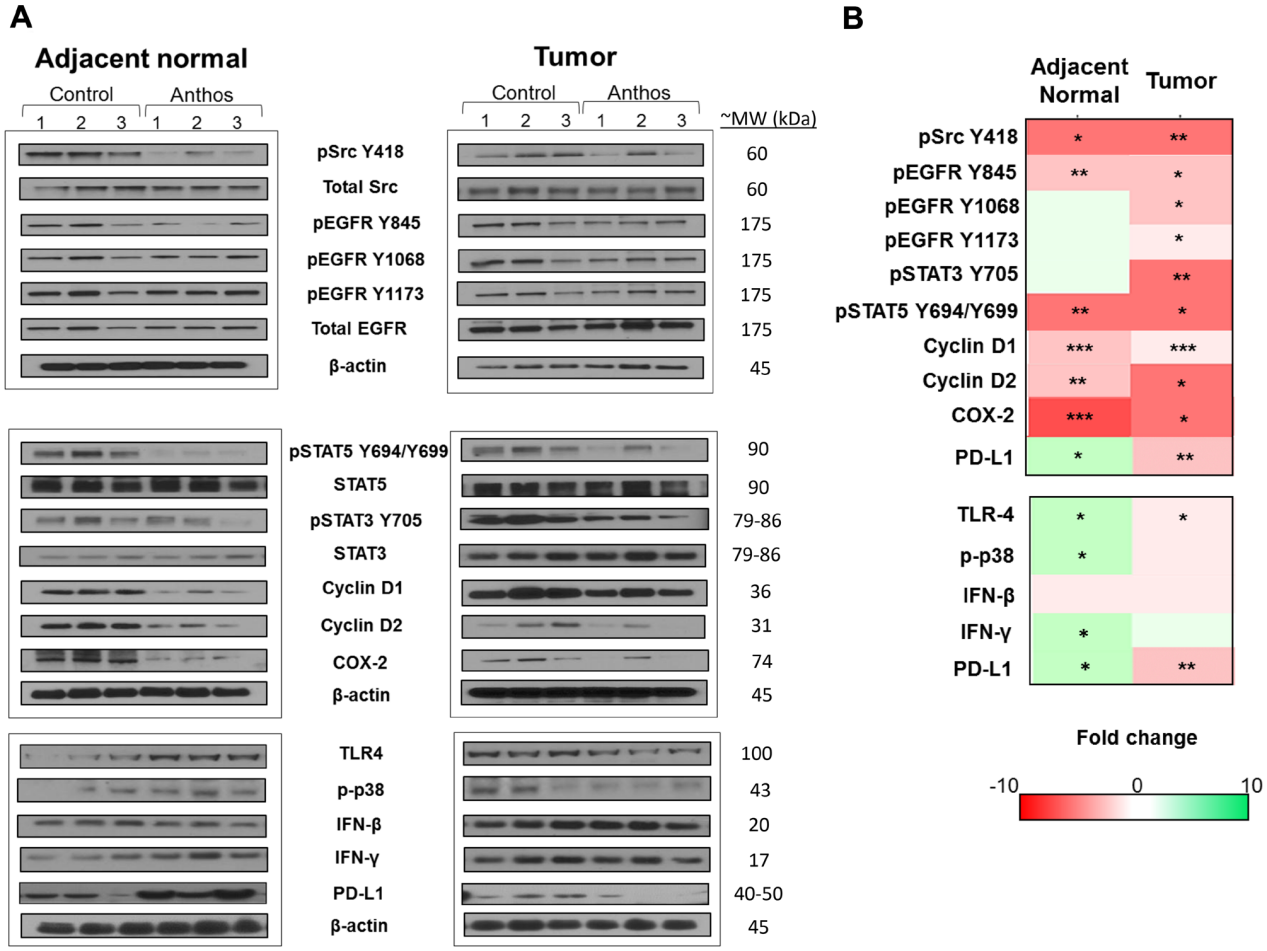
Changes in the expression of Src, EGFR and inflammatory pathways following treatment with Anthos *in vivo*. Changes in the (**A**) phosphorylation and expression of Src and EGFR along with corresponding downstream pathway targets including STAT3, STAT5, cyclin D1, cyclin D2, and COX-2 and immune related proteins TLR-4, p38, IFN-β, IFN-γ and PD-L1 in adjacent normal and tumor colon tissue. Tissue samples, taken from ETBF-inoculated *Apc^Min^*^/+^ mice after treatment with Anthos or vehicle, were analyzed by western blot, with β-actin loading control. (**B**) Representative heat maps for *in vivo* changes in Src, EGFR and inflammatory pathways. Legend depicts the average fold change and significance level in either phosphorylation status or expression levels between Anthos-treated and vehicle control animals within either adjacent normal (*n* = 3) or tumor colon tissue (*n* = 3), ^***^signifies *P* < 0.001, ^**^signifies *P* < 0.01, and ^*^signifies *P* < 0.05.

To understand Anthos mechanism of action we focused initially on the Src- and EGFR-related pathways. EGFR and Src, by way of its enhancement of EGFR signaling are key pathways in the development of colorectal cancer, along with several other cancers [31]. Our preliminary analysis of lysates prepared from Anthos-treated APC mutant HT-29 cells showed that Anthos treatment led to a dose-dependent decrease in the phosphorylation status of EGFR (Y1068) and Src (Y418) (data not shown). We then sought to determine the influence of Anthos treatment on the Src- and EGFR-related pathways in an ETBF-treated *Apc^Min^*^/+^ mouse model, which have been reported to have increased EGFR activity and c-Src expression in the adenoma and intestinal enterocyte tissue [32]. Results from our analysis of the phosphorylation status of the tyrosine kinase, Src at position Y418 showed a significant decrease in phosphorylation in Anthos-treated animals compared to control animals in tumor (*P* = 0.007) and adjacent normal colon tissue (*P* = 0.013) (Figure 5). The phosphorylation status of transphosphorylation site Y845 of the receptor tyrosine kinase (RTK), EGFR, a site noted for its activation by Src was also significantly decreased in adjacent normal (*P* = 0.003) and colon tumor (*P* = 0.01) samples taken from Anthos–treated animals over control animals. The transcription factor, STAT5a/b, which is immediately downstream of EGFR Y845, also showed a significant decrease in phosphorylation at tyrosine positions 694 and 699 in both adjacent normal (*P* = 0.004) and tumor (*P* = 0.01) tissue samples from Anthos-treated animals. The ultimate expression of the downstream targets cyclin D1, cyclin D2 and COX-2 were also significantly downregulated in the Anthos group for both adjacent normal (*P* = 0.0009, *P* = 0.001, and *P* = 0.0009, respectively) and tumor tissue samples (*P* = 0.0004, *P* = 0.03, and *P* = 0.03, respectively) (Figure 5). Interestingly, significant differences in the phosphorylation status of the autophosphorylation sites of EGFR Y1068 and Y1173 were noted in the tumor (*P* = 0.01 and *P* = 0.03, respectively) but not the adjacent normal colon tissue (*P* = 0.09 and *P* = 0.10, respectively) of Anthos treated compared to control animals. This difference between adjacent normal and tumor tissue was also noted in the phosphorylation status of the immediate downstream protein STAT3, with a significant decrease in the phosphorylation at Y705 observed only in the tumor tissue (*P* = 0.007) and not the adjacent normal (*P* = 0.19) tissue in Anthos treated animals. No significant changes in the levels of total EGFR were observed.

Building upon the histopathology findings as well as Src and EGFR findings, we next sought to determine the influence of Anthos treatment on the inflammatory tumor and adjacent normal microenvironments in the same tissue samples. Results from our western blot analysis of the inflammation pathways associated with CRC and immune-checkpoint function showed significant increases in the expression of IFN-γ (*P* = 0.04), phospho-p38 (*P* = 0.03), and levels of TLR-4 (*P* = 0.01), and significant decreases in the expression of COX-2 (*P* = 0.0009), in adjacent normal tissue (Figure 5). In the tumor tissue microenvironment, reduced levels of TLR-4 (*P* = 0.01) were noted along with reduced expression of COX-2 (*P* = 0.03) in the Anthos group compared with vehicle treatment. Interestingly, expression of the key immune-checkpoint protein, PD-L1, was significantly increased in adjacent normal tissue (*P* = 0.01) of animals treated with Anthos versus vehicle control and significantly decreased in tumor tissue in these same Anthos versus control animals (*P* = 0.005).

## DISCUSSION

Although much progress has been made in the treatment of CRC, it still remains the third leading cause of cancer-related death in the U.S. Furthermore, FAP remains an orphan disease with the only viable treatment option being surgical resection of the colon. Even after this drastic procedure, individuals with FAP are still at a greater risk for developing cancers of the small intestine. Therefore, the development of alternative prevention and treatment strategies for combating both FAP and CRC are of great importance and represent an unmet need.

One particular class of plant bioactive compounds that have been studied for their health-promoting properties are berry anthocyanins. This family of colored pigments is particularly promising given its long history as a dietary constituent for many humans. However, anthocyanins, when taken in their native form are often plagued by issues with their limited bioavailability and stability, which effectively limit their translation into a clinically-viable option due to the large doses needed in order to elicit therapeutic efficacy. With this in mind, we therefore focused on the Anthos, which we have previously shown to be more active than the native anthocyanin counterpart in A549 lung cancer cells [27]. In this paper, we also showed that this relationship is valid in colon cancer with delphinidin, and cyanidin yielding greater potency than their 3-monoglucoside and 3,5-diglucoside anthocyanin counterparts. These results are not surprising considering that anthocyanidins are somewhat more lipophilic due to their higher partition coefficients than their anthocyanin counterparts. For example, the anthocyanin, cyanidin 3,5-O-diglucoside has an octanol to water (K_OW_) partition coefficient of 0.21 as opposed to its anthocyanidin counterpart, cyanidin which has a partition coefficient of 10.07 [33]. These higher partition coefficients enable anthocyanidins to be taken up passively by cells whereas anthocyanins are known to require facilitated transport and thus may be limited in their transport efficiency/limitation [34].

Anthocyanidins were not only more potent than their anthocyanin counterparts, but also exhibited selective toxicity towards colon cancer cells over the normal colon cells with SI values that were well above the recommended cutoff for lack of toxicity. We had previously shown that native mixture of bilberry-derived Anthos were non-toxic in wild-type mice [35]. Furthermore, work with anthocyanins derived from blueberry, black currant as well as elderberry showed no toxicity when administered to rats (20 mg/kg/day), mice (25 mg/kg/day), or guinea pigs or rats (>3 g/day for 15 days or 90 days) [36]. It should be noted that prior to any clinical work being done, additional testing would need to be undertaken.

Given the lack of more favorable/less invasive treatment options for FAP and the severity and ultimate lethality of the disease at early ages, there is a great need for developing alternative preventative treatment options. With this unmet need, the potential application of Anthos to the prevention of FAP is a key possibility based on our *in vivo* findings indicating significant decreases in intestinal polyp number with Anthos intervention in *Apc^Min^*^/+^ mouse model. In addition to the promising results attained with the chemoprevention in the FAP study, Anthos exhibited significant anti-tumor activity against ETBF-induced colorectal tumors in the *Apc^Min^*^/+^ mouse model.

ETBF, a subtype of *B. fragilis* that secrets the metalloprotease enterotoxin *B. fragilis* toxin (BFT), is associated with diarrheal disease in both humans and animals as well as active inflammatory bowel disease. A study looking at the prevalence of ETBF found that 26.8% of individuals with diarrhea and 12.4% of individuals without diarrhea had stool samples that were positive for the presence of ETBF [37]. Furthermore, a recent study looking at the bacterial composition in individuals with FAP compared to normal controls found that the colonic mucosa of individuals with FAP were highly enriched with genes for the toxin, BFT, that is secreted by the ETBF bacteria [38]. In view of our encouraging findings, it is presumable that use of Anthos in human inflammatory bowel conditions and colorectal tumors associated with ETBF and FAP could be of great benefit.

We have previously shown that Anthos favorably modulate targets such as β-catenin, cyclin D1, cyclin B1, pERK, VEGF proteins, c-myc and MMP9 in lung cancer H1299 cells [25]. Furthermore, anthocyanin substitution patterns were found to lead to different cellular signaling cascades effects [^36]^. For instance, only malvidin which features methoxy groups at the 3′ and 5′ positions of the B-ring lead to inhibition of cAMP-specific phosphodiesterases (PDEs) whereas, cyanidin and delphinidin were shown to inhibit EGFR in human vulva carcinoma A431 cells [36]. In this report, we deduced mechanistic work utilizing APC mutant colon cancer cells and adjacent normal and colon tumor tissue samples from ETBF *Apc^Min^*^/+^ mouse model treated with Anthos. It was previously shown that APC deficiency was associated with an increase in EGFR activity and c-Src expression in *Apc^Min^*^/+^ mouse adenomas and intestinal enterocytes [32]. Furthermore, in addition to increasing the secretion of chloride and permeability of intestinal epithelial cells, BFT also activates STAT3 and TH17 responses and leads to increased COX-2 [39]. However, no work had been conducted to assess the impact of Anthos or anthocyanins on Src in any cancer model or Anthos on EGFR in a colorectal cancer model. *In vitro* work using the APC mutant HT-29 colon cancer cells showed a clear dose-dependent decrease in the phosphorylation status of Src (Y418) and EGFR (Y1068). Results from our survey of the Src and EGFR-related pathways show a significant decrease in Src phosphorylation (Y418), EGFR phosphorylation (Y845) and the downstream mediator STAT5a/b (Y694 and Y699), both in adjacent normal and colon tumor tissue taken from ETBF treated *Apc^Min^*^/+^ mice that received Anthos treatment. Furthermore, phosphorylation status of EGFR at Y1068 and Y1173 along with the downstream mediator STAT3 (Y705) was shown to decrease in tumor tissue in Anthos treated mice. Key regulators of cell-cycle progression and inflammation, including Cyclin D1, Cyclin D2, COX-2 were shown to be favorably modulated by Anthos, with the most dramatic reductions in expression observed in tumor tissue samples. Overexpression of COX-2 has been noted in colon tumor tissue and plays a role in the pathogenesis of FAP, ETBF and ultimately colon cancer [40, 41]. In addition to COX-2, several other key proteins involved in the inflammatory environment including TLR4, IFN-γ and PD-L1 were shown to be favorably altered in adjacent normal and colon tumor tissue. Interestingly, a key immune-checkpoint protein, PD-L1 was shown to be significantly decreased in tumor tissue but increased in adjacent normal colon tissue samples taken from Anthos-treated animals. These mechanistic findings were particularly intriguing based upon the histopathology findings which showed that Anthos-treated animals featured lymphoid aggregates in normal colon tissue adjacent to tumors as well as in normal colon tissue. These lymphoid aggregates were absent in colon tissue taken from vehicle-treated animals. Alterations in the phosphorylation status of EGFR and Src as well as downstream STAT3 in colon tumor tissues taken from the Anthos–treated, ETBF-inoculated *Apc^Min^*^/+^ mice support this decrease in PD-L1 expression, while the simultaneous increase in PD-L1 expression in adjacent normal tissue is supported by the IFN-γ modulation noted in this same tissue. This modulatory specificity, which in fact, differentially modulated PD-L1 levels in colon tumor over normal tissue, provides an interesting application for Anthos treatment to not only the prevention of offsite toxicities associated with the current standard of care, but also serves as a potential modulator of PD-L1 over-expression specific to colon tumor tissue. Overall, these findings provide novel mechanistic insight into the method by which Anthos achieve both chemopreventive and therapeutic effects against a highly relevant bacterially-driven colon cancer model.

In summary, our data provide compelling evidence for the use of a bilberry Anthos against FAP and CRC. Anthos were shown to yield greater potency and efficacy than their counterpart anthocyanins and no toxicity toward normal colon cells *in vitro*. Furthermore, *in vivo* anti-polyp and anti-tumor efficacy along with corresponding mechanistic insight suggest that Anthos lead to a potent decrease in the phosphorylation status of Src, EGFR, STAT5, STAT3 and expression of key markers for proliferation and inflammation including cyclin D1, cyclin D2, COX-2, PD-L1 as well as positive chemopreventive modulation of the inflammatory environment. Overall, results from this study offer an exciting possibility for potential treatments with the bilberry-derived Anthos for FAP and CRC in the future.

## MATERIALS AND METHODS

Anthocyanins and anthocyanidins: The anthocyanin, delphinidin 3,5-diglucoside was a kind gift from Dr. Inder P. Singh of the National Institute of Pharmaceutical Education and Research (S.A.S. Nagar, India). The individual anthocyanidins, cyanidin, delphindin, petunidin and malvidin were purchased from Chromadex (Irvine, CA, USA). The native mixture of anthocyanidins (Anthos) isolated from standardized bilberry extract, with purity of >85% was generously provided by 3P Biotechnologies, Inc. (Louisville, KY, USA).

Isolation of bilberry-derived Anthos: The native bilberry Anthos was further enriched using C18 Sep-Pak cartridges (Waters, Milford, MA, USA). Anthos were eluted in acidified (0.1% HCl) ethanol. The enriched extract was then dried using a Savant SC210A Speed-Vac (Thermo Fisher Scientific, Waltham, MA, USA) and stored under argon at −20°C. Purity was determined by HPLC-PDA-UV. Briefly, 15 μl samples were analyzed using a Shimadzu Premier C18 reverse-phase column (250 × 4.6 mm i.d., 5 μm). Mobile phase A was composed of water: formic acid: acetonitrile (87:10:3) and mobile phase B was composed of water: formic acid: acetonitrile (40:10:50). The flow rate was 0.6 ml/min and the gradient condition was 0–5 min 5% B; 5–15 min 15% B; 15–20 min 25% B; 20–30 min 35% B; 30–40 min 45% B; 40–45 min 100% B; 45–50 min 5% B. Detection of individual anthocyanidins was at 520 nm by PDA-UV and total Anthos concentration was calculated using a standard curve. The reference compounds were purchased from Chromadex (Irvine, CA, USA) and Cayman Chemical Company (Ann Arbor, MI, USA).

Cells, culture conditions and treatments: The APC wild-type HCT 116 (*ATCC*^®^ CCL-247^™^), HT-29 (*ATCC*^®^ HTB-38D^™^) colon cancer cell lines and CCD-18Co colon (ATCC^®^ CRL-1459^™^) normal cells were acquired from American Type Culture Collection (Manassas, VA, USA). HCT-116 and HT-29 cells were maintained in McCoy’s 5A medium (Gibco, Grand Island, NY, USA) supplemented with 10% FBS, 100 U/ml penicillin and 100 μg/ml streptomycin in a humidified atmosphere containing 5% CO_2_ at 37°C. CCD-18Co cells were maintained in MeMα (Gibco, Grand Island, NY) supplemented with 20% FBS, 100 U/ml penicillin and 100 μg/ml streptomycin in a humidified atmosphere containing 5% CO_2_ at 37°C.

### Measurement of cell viability

The cytotoxicity of individual anthocyanins and anthocyanidins and the Anthos in colon cancer and normal cell lines was assessed by enzymatic reduction of the tetrazolium dye MTT. Briefly, 3.0 × 10^3^ cells/well were grown in 96-well tissue culture plates. Twenty-four h after seeding, cells were treated with varying concentrations of test agents or vehicle. After 72 h of the treatment, cells were incubated with 5 mg/ml MTT reagent for 2 h. The resulting formazan crystals were then solubilized in dimethyl sulfoxide and spectrophotometrically measured at 570 nm (Bio-rad, Philadelphia, PA). IC_50_ values were then determined using Calcysyn software version 2.1 (Biosoft, Cambridge, England).

### Western-blot analysis

For western-blot analysis, 50 μg of protein from *in vivo* tissue lysates were resolved by gel electrophoresis and electrotransferred to polyvinylidene difluoride membranes by semi-dry transfer (Biorad Trans-blot SD, Hercules, CA, USA). Blots were blocked with 4% dry powder milk or BSA for 1 h and then incubated with primary antibodies for p-EGFR (Y845), p-EGFR (Y1068), p-EGFR (Y1173), total EGFR, p-Src (Y418), total Src, pSTAT3 (Y705), total STAT3, pSTAT5 (Y694/Y699), total STAT5, COX-2, Cyclin D1, and Cyclin D2, IFN-γ, TLR-4, phospho-p38, IFN-β. These antibodies were all acquired from Santa Cruz Biotech (Santa Cruz, CA, USA); PD-L1 was acquired from Proteintech (Rosemont, IL). Blots were incubated with primary antibodies at 4°C overnight and after washing were incubated for 1 h at room temperature with respective secondary antibodies conjugated to peroxidase (Santa Cruz Biotechnology, Dallas, TX, USA). Blots were then developed with an ECL detection system. Densitometric analysis was performed using ImageJ 1.x software [42].

### Animal model for FAP and CRC

*Apc^Min^*^/+^ [43, 44] is a well-established and accepted model for studying FAP and CRC. The mice characteristically exhibit a germline nonsense mutation at codon 850 of the APC gene that causes the spontaneous development of polyps, which predominantly occur in the small intestine by the age of 10–12 weeks. In the absence of ETBF, *Apc^Min^*^/*+*^ mice rarely develop tumors in the large intestine [39].

### ETBF

Enterotoxigenic *Bacteriodes fragilis* (ETBF) exists asymptomatically in 12.4% of individuals overall and in 27% of individuals with diarrhea symptoms [37]. Furthermore, presence of ETBF in the gut is a well-known global cause of diarrheal disease that is accompanied by colitis in both humans and animals. The pathogenicity associated with ETBF is due to the secretion of a 20 kDa zinc-dependent metalloprotease toxin, *B. fragilis* toxin (BFT), which binds to colonic epithelial cells and leads to the cleavage of the tumor suppressor protein, E-cadherin, and the secretion of interleukin-8 [45]. Overall, this process leads to the stimulation of proliferation and migration of human colon cancer cells [46]. BFT has also been shown to induce pro-inflammatory cytokine secretion by further activating the NF-ƘB pathway [46].

### *In vivo* FAP and CRC studies

Animal experiments were performed in agreement with an approved protocol by the Institutional Animal Care and Use Committee at the University of Louisville. Breeding colonies were established by Dr. Nejat K. Egilmez’s lab [47] at the University of Louisville using C57BL/6J Min/+ (*Apc^Min^*^/+^) mice that were originally obtained from Jackson Laboratories (Bar Harbour, ME). Mice were genotyped for the APC mutation using PCR according to the protocol established by Jackson Laboratories. Mice were fed a standard chow diet. Animals received diet and water *ad libitum* and were maintained on a standard light/dark cycle for the duration of the study. Animals were euthanized at 13–14 weeks. Following fixation of intestines using formalin, intestinal polyps were counted using a microscope (Leica EZ4, Wetzlar, Germany) by four different experienced individuals after blinding the samples.

### FAP study dosage regimen

For the FAP study, treatments began when animals were 8–9 weeks old and were in the early disease stage. Male (*n* = 4) and female (*n* = 4) *Apc^Min^*^/+^ mice were administered by oral gavage 40 mg/kg Anthos or vehicle control (200 μl total volume per dose, per mouse, 10% DMSO in water). Animals were treated on Mondays, Wednesdays and Fridays every week for 5 weeks.

### CRC studies

Three CRC studies were performed to test the impact of the Anthos on tumor numbers. At 5–6 weeks of age, animals were administered antibiotic (clindamycin (0.1 g/L) and streptomycin (5 g/L)). Four days later, the animals were administered ETBF to promote tumorigenesis and one week following ETBF inoculation, animals began their respective treatment regimen. Animals were culled in the fed state at 12 weeks, colon tumors were counted and tissues were harvested.

### CRC dosage regimen

In the first study male *Apc^Min^*^/+^ mice were administered by gavage 20 mg/kg Anthos or vehicle control. Animals were treated 5 times a week (Monday–Friday) for 4 weeks. Animals were culled in the fed state at 12 weeks and colon tumors were counted. For the second study female *Apc^Min/^*^+^ mice were administered by gavage 40 mg/kg Anthos or vehicle control. Animals were treated 3 times a week (Monday, Wednesday and Friday) for 4 weeks. Animals were culled in the fed state at 11–12 weeks and colon tumors were counted. In the third study, we tested three different doses of Anthos: low (20 mg/kg, three doses a week, ≈8.6 mg/kg/day), medium (40 mg/kg, three doses a week, ≈17.1 mg/kg/day) and high (80 mg/kg, three doses a week, ≈34.3 mg/kg/day). Male and female *Apc^Min^*^/+^ mice (*n* = 5 combined) were randomly assigned to each treatment or vehicle control group and were treated via oral gavage three times per week (Monday, Wednesday and Friday) for 4 weeks.

### Pathology

Following excision, colon tumor and mesenteric lymph tissue were immediately fixed in a solution of 10% formalin. After 48 h, samples were transferred to a solution of 70% ethanol. Samples were embedded in paraffin and sectioned by the Pathology Core Research Laboratory, Department of Pathology and Laboratory Medicine at the University of Louisville. Samples were sectioned at 4 micron and stained with H&E and subsequently evaluated by Dr. Mostafa Fraig, an expert pathologist, who was blinded to the sample ID/treatment groups. Images were captured using an Olympus DP72 camera mounted on EX 41 microscope (Olympus Corporation, Tokyo, Japan). The sections were evaluated for the presence or absence of tumor, number of tumor foci and size of tumor, if present. Additionally, tumor grade and presence of lymphoid infiltrate near the tumor were recorded.

### Data analysis

Statistical analysis was performed using Graph Pad Prism statistical software version 4.03 (La Jolla, CA, USA) and RStudio software version 1.0.153 (Boston, MA, USA) Lattice package [48, 49]. Test for normality, *F*-test for equal variance, and appropriate *t*-test were used for the first two animal studies, and for analysis of western blot results. For the third animal study (the dose escalation study) we used one-way ANOVA and Tukey post-hoc test analysis. IC_50_ values were determined using CalcuSyn software version 2.1 (Biosoft, Cambridge, England). Heat maps were constructed using RStudio software version 1.0.153 (Boston, MA, USA) gplot package [49, 50].

## Abbreviations

CRC: colorectal cancer
Anthos: native anthocyanidins mixture from billberry
FAP: familial adenomatous polyposis
APC: adenomatous polyposis coli
COX-2: cyclo-oxygenase-2
Cy: cyanidin
Dp: delphinidin
Pt: petunidin
Mv: malvidin
Pe: peonidin
HPLC: high performance liquid chromatography
PDA: photodiode array detector
UV: ultraviolet
ETBF: enterotoxigenic Bacteriodes *fragilis*
BFT: *B. fragilis* toxin
